# Supplementary material for: Skyrmions based on optical anisotropy for topological encoding
Source: Light Sci Appl. 2026 May 27;15:254. doi: 10.1038/s41377-026-02307-4 (PMC13213056; doi:10.1038/s41377-026-02307-4)
Supplement: Supplementary file 1 — Supplementary Information for Skyrmions based on optical anisotropy for topological encoding [file 41377_2026_2307_MOESM1_ESM.pdf]

Supplementary Information for

Skyrmions based on optical anisotropy for topological encoding

Yunqi Zhang[1,†], An Aloysius Wang[1,†], Runchen Zhang[1,†], Zimo Zhao[1], Yifei Ma[1], Ruofu Liu[1], Zhi-Kai Pong[1], Yuxi Cai[1], and Chao He[1,*]

[1]Department of Engineering Science, University of Oxford, Parks Road, Oxford, OX1 3PJ, UK
[*]Corresponding authors: chao.he@eng.ox.ac.uk

1 Mueller matrix measurement and decomposition

In our experiments, Mueller matrices are characterized using a dual-rotating-retarder Mueller matrix polarimeter. This system consists of a polarization state generator (PSG) and a polarization state analyser (PSA) [1], each implemented with a rotating quarter waveplate (QWP) and a horizontal polarizer. Each Mueller matrix element was obtained by rotating two QWPs and recording the corresponding intensity measurements, followed by reconstruction using the algorithm described in [2]. The rotation speeds of the two QWPs were optimized to minimize noise propagation throughout the measurement process [3]. Calibration of the dual-rotating-retarder Mueller matrix polarimeter is likewise performed using Fourier analysis to correct systematic errors inherent to the experimental setup [4].

We further detail the implementation of the Mueller matrix polar decomposition (MMPD) originally proposed by Lu and Chipman [5], which is implicitly used in the definition of $\mathcal{P}_{\text{aniso}}$. This approach decomposes Mueller matrices to systematically extract diverse polarization characteristics. Specifically, the method factorizes a general Mueller matrix into the sequential product of three matrices: a depolarizer matrix $\boldsymbol{M_\Delta}$, a retarder matrix $\boldsymbol{M_R}$, and a diattenuator matrix $\boldsymbol{M_D}$. Given that our focus is strictly on structured matter behaving purely as a retarder, the synthesized Mueller matrix simplifies accordingly and is expressed as follows:

$$\boldsymbol{M} = \boldsymbol{M}_R = \begin{bmatrix} 1 & 0 & 0 & 0 \\ 0 & m_{22} & m_{23} & m_{24} \\ 0 & m_{32} & m_{33} & m_{34} \\ 0 & m_{42} & m_{43} & m_{44} \end{bmatrix} = \begin{bmatrix} 1 & \boldsymbol{0}^{\mathrm{T}} \\ \boldsymbol{0} & \boldsymbol{m}_R \end{bmatrix} \tag{1}$$

The Mueller matrix encapsulates both the axis geometry and the retardance, as represented in equation (1). These two key polarization parameters are extracted as follows:

$$\theta_s = \cos^{-1}\left[\frac{\mathrm{tr}(\boldsymbol{M}_R)}{2} - 1\right] = \cos^{-1}\left[\frac{1+\mathrm{tr}(\boldsymbol{m}_R)}{2} - 1\right] = \cos^{-1}\left[\frac{\mathrm{tr}(\boldsymbol{m}_R) - 1}{2}\right] \tag{2}$$

$$A_i = \frac{1}{2\sin\theta_s} \sum_{j,k=1}^{3} \epsilon_{ijk} (\boldsymbol{m}_R)_{jk} \tag{3}$$

where θ_s denotes the retardance, ϵ_{ijk} is the Levi-Civita symbol, and $A_i, i = 1, 2, 3$ represent the components of the axis geometry $\boldsymbol{A} = [A_1\ A_2\ A_3]^{\mathrm{T}}$. To uniquely determine the axis geometry, accurate characterization of the sample's Mueller matrix is essential. An important ambiguity must be addressed. Any given retarder, whose retardance ranges from 0 to 2π, has a corresponding retarder with different parameters but the same Mueller matrix. This corresponding retarder has a fast axis orthogonal to that of the original (i.e., aligned with its slow axis) and a retardance equal to 2π minus that of the original retarder. In this work, we fix the retardance value at 0.5π for all designed patterns to avoid ambiguity in the decomposition and ensure a unique solution. To illustrate the practical extraction of these parameters from the Mueller matrix, we present two representative examples based on numerical simulations, as shown in Figure S1.

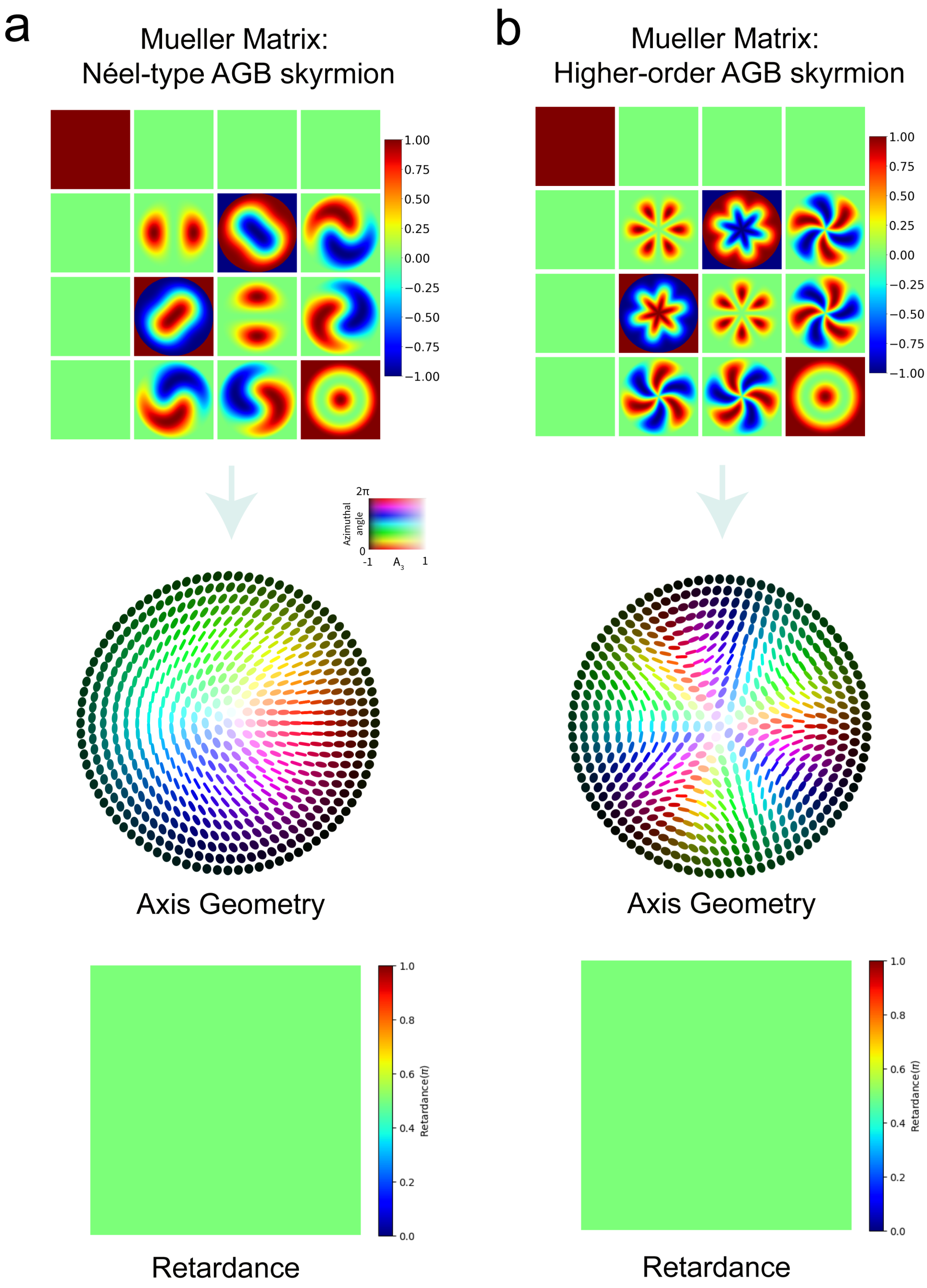

Figure S1: Mueller matrix decomposition of AGB skyrmions. Decomposition of Mueller matrices illustrating two representative examples of AGB skyrmions. **a.** Mueller matrix of a Néel-type skyrmion, together with its corresponding axis geometry and retardance maps, highlighting the uniform retardance and spatially varying axis geometry. The axis geometry field is depicted with hue indicating the azimuthal angle and saturation representing the height (see Figure 1 in the main text). **b.** Mueller matrix of a higher-order skyrmion, showcasing complex axis geometry distribution and uniform retardance. The retardance for both examples is fixed at $\theta_s = 0.5\pi$.

2 Experimental setup and skyrmion pattern design

In our experiments, we employed three cascaded LC-SLMs to precisely manipulate synthesized arbitrary elliptical retarders, thereby generating AGB skyrmions. To characterize these skyrmions comprehensively, we utilized a PSG and a PSA, enabling accurate Mueller matrix measurements. The complete experimental setup and the parameters of the devices are detailed in Figure S2. In practice, we first design the Mueller matrix of the AGB-LC skyrmion according to the spatially varying axis geometry and retardance. This Mueller matrix was then decomposed using the method in [6] and separately loaded onto three LC-SLMs. An example of LC-SLM patterns for a Néel-type skyrmion and their Mueller matrices are illustrated in Figure S3.

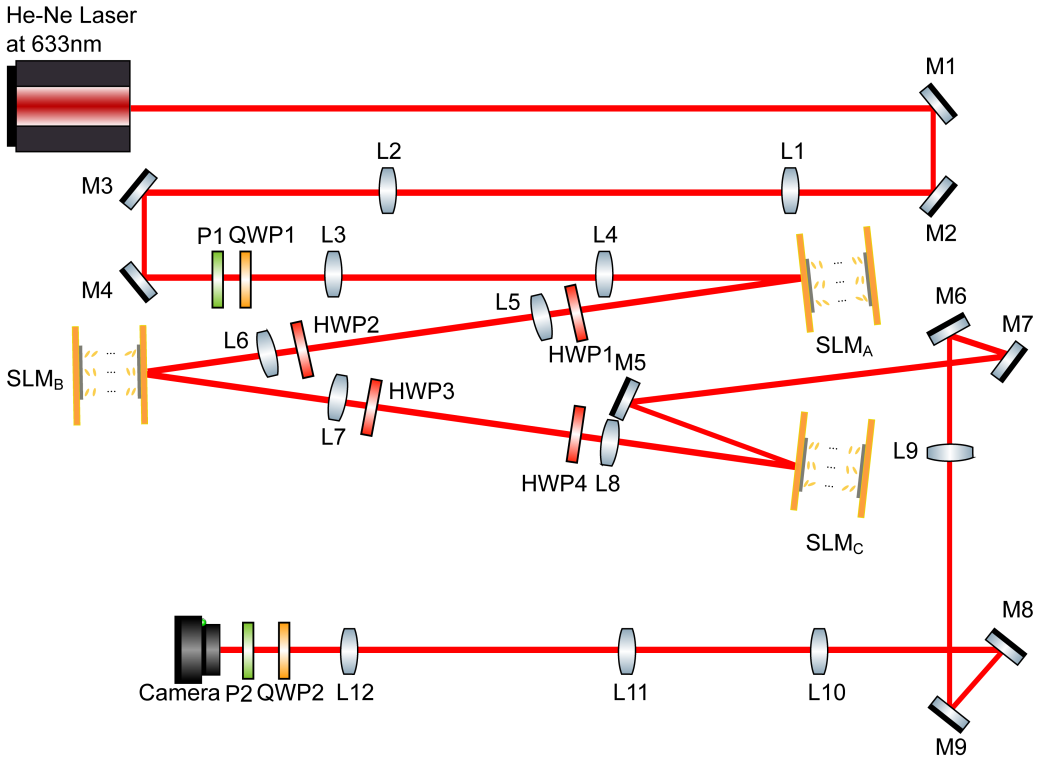

Figure S2: Schematic diagram of the experimental setup. The experimental setup. The laser source is a He-Ne laser operating at 633 nm (Melles Griot, 05-LHP-171, 632.8 nm). Mirrors M1-M9 are silver mirrors (Thorlabs, PF03-03-P01). Lenses L1-L12 are achromatic doublet lenses. L1 and L2 expand the beam, while L3-L10 relay it. L11 and L12 are used to image the beam onto the camera. P1 and P2 are polarizers (Thorlabs, GL10-A); QWP1 and QWP2 are quarter-wave plates (Thorlabs. WPQ10M-633). Together, they form the PSG and PSA, respectively. SLM_A, SLM_B, and SLM_C are three LC-SLMs (Hamamatsu, X10468-01). HWP1-HWP4 are half-wave plates (Thorlabs, WPH10M-633) used to orient SLM_B at a diagonal angle. Images are captured and saved by a camera (Thorlabs, DCC3240N).

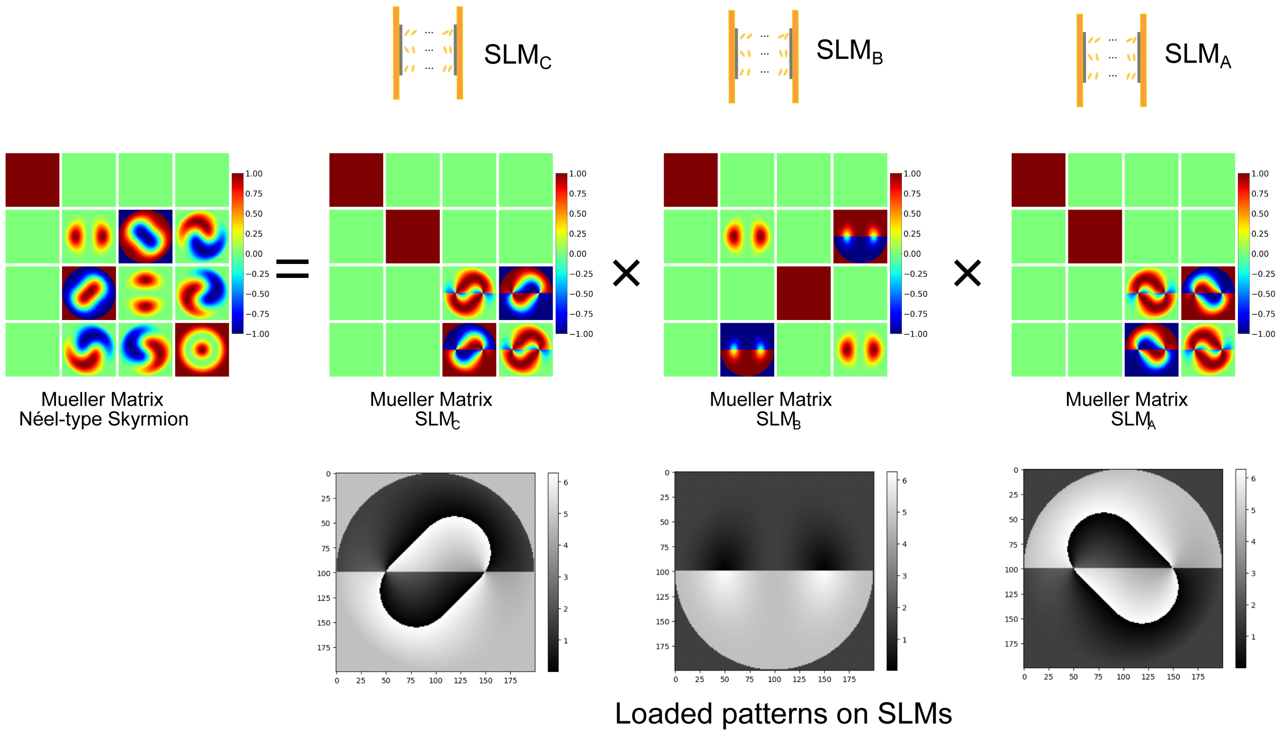

Figure S3: Examples of loaded patterns on LC-SLMs and corresponding Mueller matrices. Illustrations of patterns loaded onto three cascaded LC-SLMs, labelled as SLM$_A$, SLM$_B$, and SLM$_C$. Each pattern comprises 200 $\times$ 200 pixels, and the LC-SLM has a pixel pitch of 12.5 μm. The Mueller matrix of the resulting Néel-type skyrmion (left) is achieved by multiplying the Mueller matrices generated from each individual SLM pattern sequentially. Similar methods can be applied to construct various other skyrmion structures.

3 The definitions of AGB skyrmions

In this section, we follow the conventions of [7] and begin by recalling the definition of the topological degree [8]. Consider a smooth map $f\colon M \longrightarrow N$ between compact, connected, and oriented manifolds of the same dimension. The degree, expressed in terms of differential forms, is:

$$\int_M f^*\Omega = \deg f \int_N \Omega \tag{4}$$

where Ω is a top form whose integral is non-zero, $f^*\Omega$ denotes its pull-back, and $\deg f$ is the degree of map f. For a skyrmion, the target manifold N is the two-sphere S^2 (for example, in polarization optics, this corresponds to the Poincaré sphere) and the form Ω is typically taken to be the Riemannian volume form of the standard metric on S^2.

In order for a planar field to be a skyrmion, the domain must also be a compact, connected, and oriented manifold. One approach to achieve this is through the following compactification procedure. For a planar field $\boldsymbol{S} : U \to S^2$, where U here is an open subset of the plane $\mathbb{R}^2$, suppose there exists a connected, oriented and compact manifold M and a smooth map $\varphi : U \to M$ satisfying the following properties.

1. φ is an orientation preserving diffeomorphism of U onto its image,
2. $\varphi(U)$ is a dense subset of M with full measure,
3. $\boldsymbol{S} \circ \varphi^{-1}$ extends via continuity to a smooth map $\tilde{\boldsymbol{S}}$ on all of M.

we then call $\boldsymbol{S}$ compactifiable and denote $\tilde{\boldsymbol{S}}$ as its compactification, assigning the field $\boldsymbol{S}$ a degree defined by $\deg \boldsymbol{S} := \deg \tilde{\boldsymbol{S}}$. Specifically, letting x and y be the coordinate functions on U, the skyrmion number can then be derived as:

$$\deg \boldsymbol{S} := \int_M \tilde{\boldsymbol{S}}^*\Omega = \int_U \varphi^*(\tilde{\boldsymbol{S}}^*\Omega) = \int_U (\tilde{\boldsymbol{S}} \circ \varphi^*)\Omega = \int_U \boldsymbol{S}^*\Omega = \frac{1}{4\pi} \iint_U \boldsymbol{S} \cdot \left(\frac{\partial \boldsymbol{S}}{\partial x} \times \frac{\partial \boldsymbol{S}}{\partial y} \right) dxdy \tag{5}$$

equation (5) is the skyrmion integral used in this work, and in our case, the manifold U is chosen to be an open unit disc.

4 Sufficient condition for AGB skyrmions topological protection

Here we detail the 60° criterion. Suppose $\boldsymbol{A} : U \to S^2$ is the axis geometry field. Let $\mathcal{P} : \mathbb{R}^3 \setminus \{(0,0,0)\} \to S^2$ be the projection defined by

$$\mathcal{P}(\boldsymbol{\xi}) = \frac{1}{\|\boldsymbol{\xi}\|}\,\boldsymbol{\xi} \tag{6}$$

where $\boldsymbol{\xi}$ is an arbitrary vector. Let the perturbed axis geometry field $\boldsymbol{A}'$ be written in the form

$$\boldsymbol{A}' = \mathcal{P}(\boldsymbol{A} + \boldsymbol{\delta}) \tag{7}$$

In Methods 2, we derive that the skyrmion number of an AGB skyrmion is topologically protected if $\|\boldsymbol{\delta}\| < 1$. For any $\boldsymbol{A}'$ defined by equation (7) with $\|\boldsymbol{A}' - \boldsymbol{A}\| < 1$, there exists a perturbation $\boldsymbol{\delta} = \boldsymbol{A}' - \boldsymbol{A}$ satisfying $\|\boldsymbol{\delta}\| < 1$. Therefore,

$$\|\boldsymbol{A}' - \boldsymbol{A}\|^2 = \|\boldsymbol{A}'\|^2 + \|\boldsymbol{A}\|^2 - 2\,\boldsymbol{A}' \cdot \boldsymbol{A} = 2 - 2\cos\alpha \tag{8}$$

where $\|\boldsymbol{A}'\| = \|\boldsymbol{A}\| = 1$ and α is the angle between $\boldsymbol{A}'$ and $\boldsymbol{A}$. To satisfy $\|\boldsymbol{A}' - \boldsymbol{A}\| < 1$, we require $\frac{1}{2} < \cos\alpha < 1$ and $\alpha < 60°$. Geometrically, if the axis geometry at a given spatial point is represented by a vector $\boldsymbol{A}$ on the axis geometry sphere, then all perturbations satisfying $\|\boldsymbol{\delta}\| < 1$ lie within a sphere of unit radius centered at the endpoint of $\boldsymbol{A}$. As illustrated in Figure S4, the maximum angular separation between $\boldsymbol{A}$ and any admissible perturbed state $\boldsymbol{A}'$ occurs when the corresponding vectors form an equilateral triangle, yielding a maximal angular deviation of 60°.

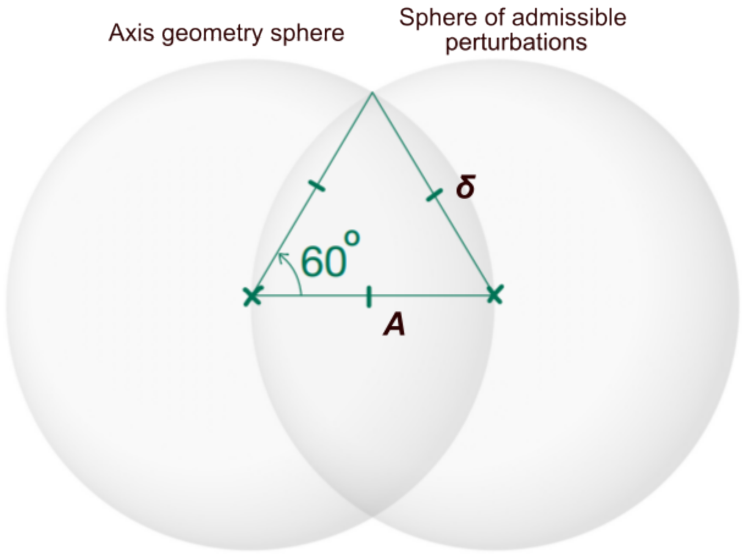

Figure S4: Geometric interpretation of the 60° condition. An axis geometry vector $\boldsymbol{A}$ on the axis geometry sphere is shown together with the sphere of admissible perturbations $\boldsymbol{\delta}$ satisfying $\|\boldsymbol{\delta}\| < 1$. The maximum angular separation between $\boldsymbol{A}$ and $\boldsymbol{A}'$ occurs when the vectors form an equilateral triangle, as indicated, yielding a maximal angular deviation of 60°.

5 Character and image encoding with AGB skyrmions

Here, we demonstrate how AGB skyrmions can be utilized for information storage. In the main text, we experimentally show that skyrmion bags can be used to encode characters, thus enabling memory applications. The encoding rules used are defined as follows: the reading sequence is determined by the chirality of the enclosing skyrmion (counterclockwise in our experiments). Once a starting reference point is selected, the reading sequence proceeds from the rightmost internal skyrmion and continues sequentially (as shown in Figure S5, following the order $(\alpha, \beta, \gamma, \delta)$). Each skyrmion within the bag is assigned one of the values $\{-2, -1, 1, 2\}$. Every two skyrmions form one byte: the first skyrmion represents the high nibble (four bits) of the hexadecimal code, while the second skyrmion represents the low nibble. This enables ASCII character storage using AGB skyrmions. An example of the first ASCII character 'NUL' is shown in Figure S5.

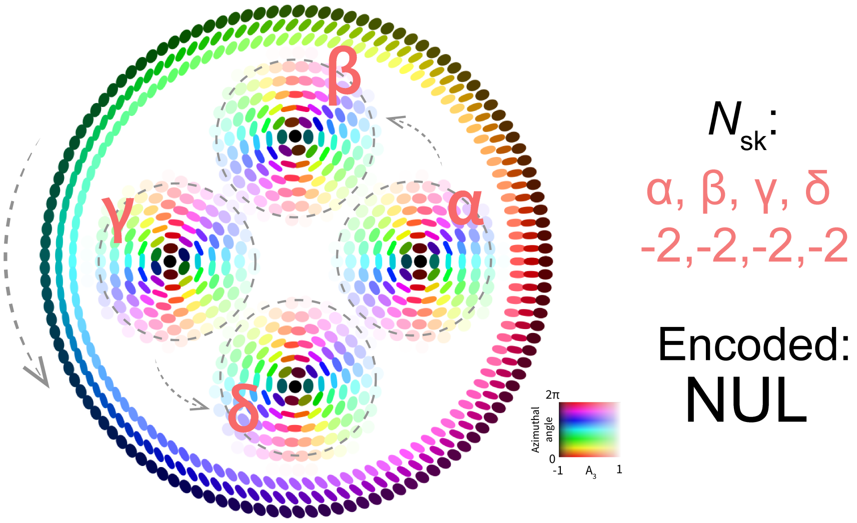

Figure S5: Illustration of the AGB skyrmion-based encoding principle using skyrmion bags. The four enclosed skyrmions (labeled α, β, γ, δ) possess skyrmion numbers of -2, -2, -2, and -2. When read sequentially in a counterclockwise direction following the encoding convention, these numbers translate to the ASCII character 'NUL'.

Based on this encoding strategy, we establish a complete ASCII encoding table, as presented in Figure S6.

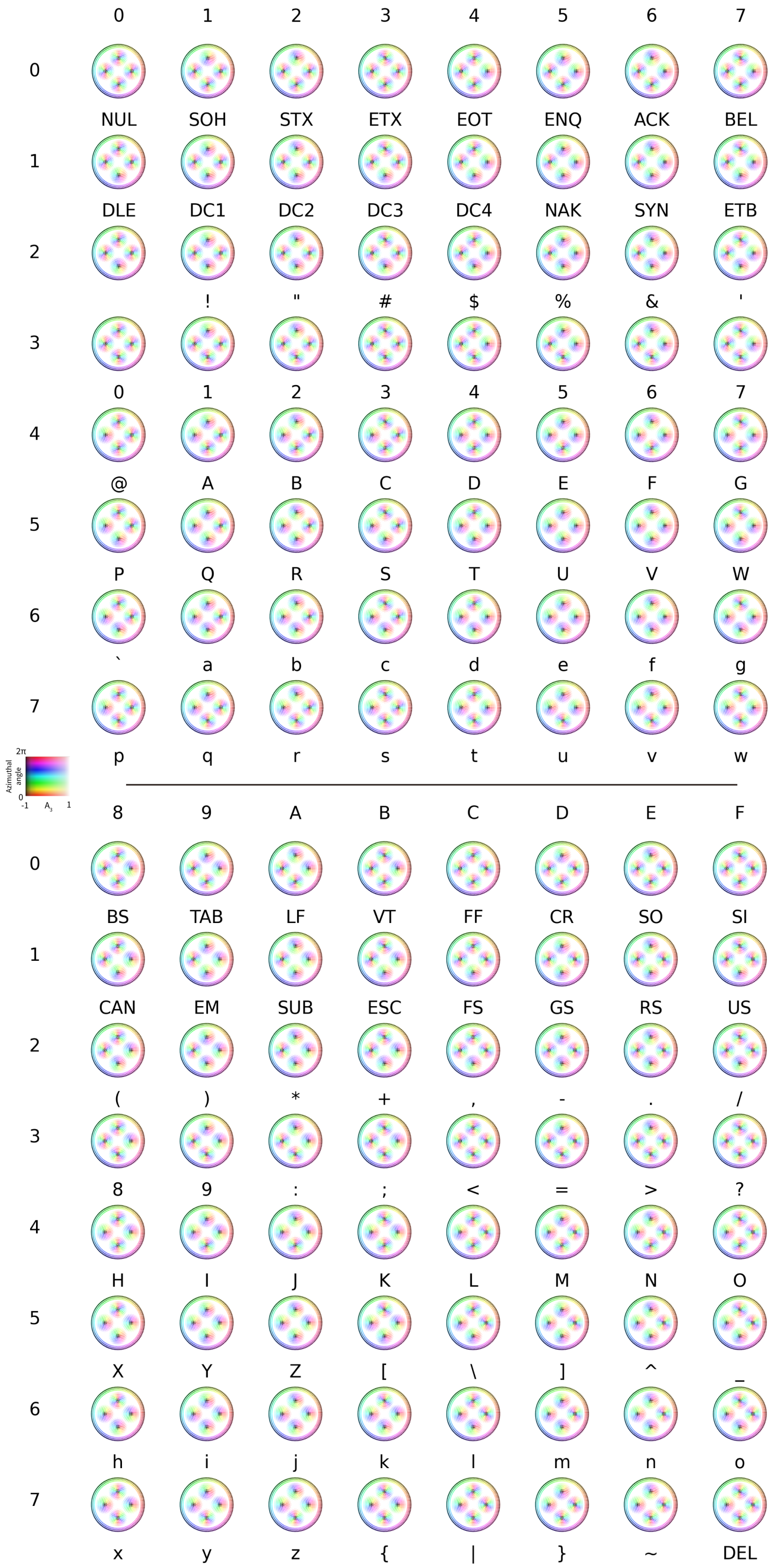

Figure S6: Complete ASCII encoding table using AGB skyrmion bags. Each pattern is labeled below with its corresponding ASCII character.

Furthermore, we demonstrate an illustrative example of image storage enabled by a lattice of skyrmions. In principle, since the number of skyrmions is unbounded, one can realize storage of an arbitrary number of bits in a single skyrmion. However, with practical considerations such as camera resolution and feature fabrication size, we employ a 4-bit grayscale scheme to encode the VOP (Vectorial Optics and Photonics) group logo [9]. The emblem is first converted to grayscale and resampled into a two-dimensional array comprising four discrete intensity levels. Each pixel is then mapped to an AGB skyrmion, yielding an ordered skyrmion array. By integrating the local skyrmionic texture, we obtain the skyrmion numbers and reconstruct the image exclusively from these discrete topological charges (see Figure S7). The demonstration involves 400,000 pixels and correspondingly, 10,000 individual skyrmions. Nanophotonic devices such as metasurfaces and laser-written retarder plates can offer subwavelength feature sizes: a single AGB skyrmion can be confined to less than 20 μm. If each AGB skyrmion encodes 10 bits and occupies a volume of 20 $\mu m \times 20$ $\mu m \times 0.5$ $\mu m \approx 200$ μm^3, then the corresponding information density reaches $\approx 5 \times 10^{16}$ bits m^{-3}. Furthermore, beyond LCs and nanophotonic devices, other material platforms that provide spatially varying birefringence can also form AGB skyrmions and may contribute to a much higher storage density. Collectively, these attributes point to a high-density, robust platform underpinned by skyrmionic topology that can be used for information storage.

At the present stage, the primary factors limiting the practical application of skyrmion-lattice-based information storage methods lie in the need for accurate and fast measurements of the device, which are required to reconstruct skyrmions and to compute their corresponding skyrmion numbers. Specifically, first, as shown in Figure S7, storing high-resolution images requires a high density of skyrmions. When the device size is fixed, this typically implies a reduction in the size of each individual skyrmion, or equivalently, each pixel. It should be noted that experimental measurements are always based on discrete sampling rather than continuous fields. Investigating the topological properties of discretely sampled fields is therefore an interesting and important problem, and one that we are actively working on.

Second, the efficient computation of skyrmion number tuples for image reconstruction also constitutes a limitation. Although this task remains challenging, a number of approaches have been proposed, ranging from analytical strategies to methods based on artificial intelligence. We anticipate that, as the field develops, more reliable and faster techniques for retrieving skyrmion numbers from numerical data will be established, thereby enabling the use of AGB skyrmions as a practical platform for information storage.

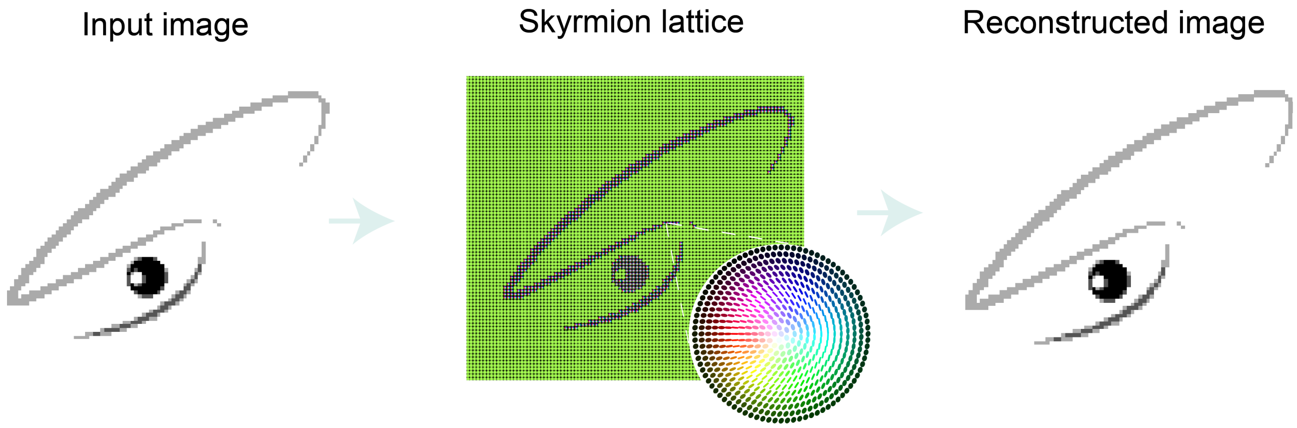

Figure S7: Skyrmion lattice for image storage and reconstruction. The input image is first converted into a 4-bit grayscale intensity map. Each pixel is then encoded by an AGB skyrmion, where the skyrmion number corresponds to the quantized intensity level of the pixel. The skyrmion numbers are subsequently evaluated and mapped back to their respective intensity levels to reconstruct the image. This approach demonstrates the potential of skyrmion lattices as a high-density, topologically protected medium for data storage and retrieval.

References

[1] RMA Azzam. Photopolarimetric measurement of the mueller matrix by fourier analysis of a single detected signal. *Optics Letters*, 2(6):148–150, 1978.

[2] Dennis H Goldstein. Mueller matrix dual-rotating retarder polarimeter. *Applied optics*, 31(31):6676–6683, 1992.

[3] Matthew H Smith. Optimization of a dual-rotating-retarder mueller matrix polarimeter. *Applied optics*, 41(13):2488–2493, 2002.

[4] Dennis H Goldstein and Russell A Chipman. Error analysis of a mueller matrix polarimeter. *Journal of the Optical Society of America A*, 7(4):693–700, 1990.

[5] Shih-Yau Lu and Russell A Chipman. Interpretation of mueller matrices based on polar decomposition. *JOSA A*, 13(5):1106–1113, 1996.

[6] Chao He, Binguo Chen, Zipei Song, Zimo Zhao, Yifei Ma, Honghui He, Lin Luo, Tade Marozsak, An Aloysius Wang, Rui Xu, et al. A reconfigurable arbitrary retarder array as complex structured matter. *Nature Communications*, 16(1):4902, 2025.

[7] An Aloysius Wang, Zimo Zhao, Yifei Ma, Yuxi Cai, Runchen Zhang, Xiaoyi Shang, Yunqi Zhang, Ji Qin, Zhi-Kai Pong, Tádé Marozsák, et al. Topological protection of optical skyrmions through complex media. *Light: Science & Applications*, 13(1):314, 2024.

[8] Boris A Dubrovin, Anatolij Timofeevič Fomenko, and Serge Petrovich Novikov. *Modern geometry—methods and applications: Part II: The geometry and topology of manifolds*, volume 104. Springer Science & Business Media, 2012.

[9] VOPLab. Voplab. `https://voplab.com/`, 2025. Accessed: 2025-07-15.
